# Acute corneal hydrops during pregnancy with spontaneous resolution after corneal cross-linking for keratoconus: a case report

**DOI:** 10.1186/s13256-017-1201-y

**Published:** 2017-02-25

**Authors:** Ricardo Alexandre Stock, Thaís Thumé, Elcio Luiz Bonamigo

**Affiliations:** Universidade do Oeste de Santa Catarina, Rua Getúlio Vargas, 2125, Bairro Flor da Serra, 89600-000 Joaçaba, Santa Catarina Brazil

**Keywords:** Cornea, Keratoconus, Corneal edema, Corneal opacity, Corneal topography

## Abstract

**Background:**

Keratoconus may progress to acute corneal hydrops even after cross-linking. In some cases, keratoconus progresses during pregnancy. In this report, we present a case of a patient with increased anterior stromal resistance after cross-linking that would favor nonprogression of keratoconus during pregnancy.

**Case presentation:**

We report that cross-linking is likely to have had a protective effect in a white pregnant patient with acute corneal hydrops who showed rapid improvement, as documented by corneal topography. Improvement occurred within 8 days, whereas up to 250 days are reported in the literature. No keratoconus progression occurred in the 20-month follow-up period.

**Conclusions:**

Cross-linking failed to prevent the occurrence of acute corneal hydrops after rupture of Descemet’s membrane but most likely helped to accelerate the resolution of the condition. Corneal hardening resulting from cross-linking may have also contributed to stabilizing keratoconus during pregnancy.

## Background

Keratoconus is a multifactorial, noninflammatory degeneration of the cornea that causes a loss of stability. It is clinically characterized by central thinning of the cornea and irregular astigmatism, which reduce visual acuity (VA). The treatment for keratoconus depends on its severity, and corneal collagen cross-linking (CXL) is an excellent treatment option in cases of disease progression [1]. We report a case of a pregnant patient who progressed to acute corneal hydrops after completing CXL treatment with an unusual resolution that occurred in 8 days without scarring. Resolution normally occurs within 5 to 36 weeks with scarring [2].

## Case presentation

**Fig. 1:**
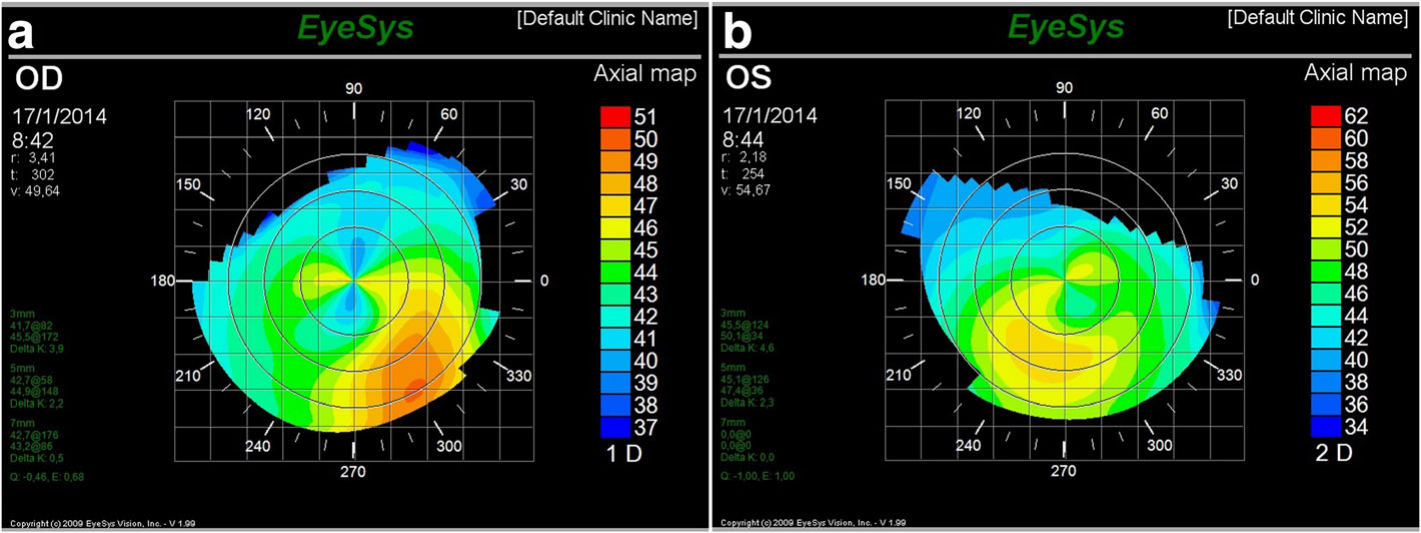
Preoperative corneal topography RE (**a**) and LE (**b**)

A 26-year-old white woman without systemic disease presented with bilateral progressive keratoconus, showing best-corrected VAs of 20/30 and 20/40 in the right eye (RE) and left eye (LE), respectively. Corneal topography showed maximum corneal curvatures of 49.64 and 54.67 diopters in the RE and LE, respectively (Fig. 1a and b), with central corneal thicknesses measured by ultrasonic pachymetry of 471 and 484 μm for the RE and LE, respectively.

The patient was subjected to standard protocol CXL in both eyes and showed best-corrected VAs of 20/30 and 20/40 in the RE and LE, respectively, 3 months after the procedure. Biomicroscopy showed a demarcation line in the anterior stroma without haze.

The patient began using rigid contact lenses (RCLs) 5 months after the procedure and reached a VA of 20/20. The patient returned complaining of pain, photophobia, and low VA in her LE 7 months after undergoing CXL, when she mentioned her 5-month pregnancy.

**Fig. 2:**
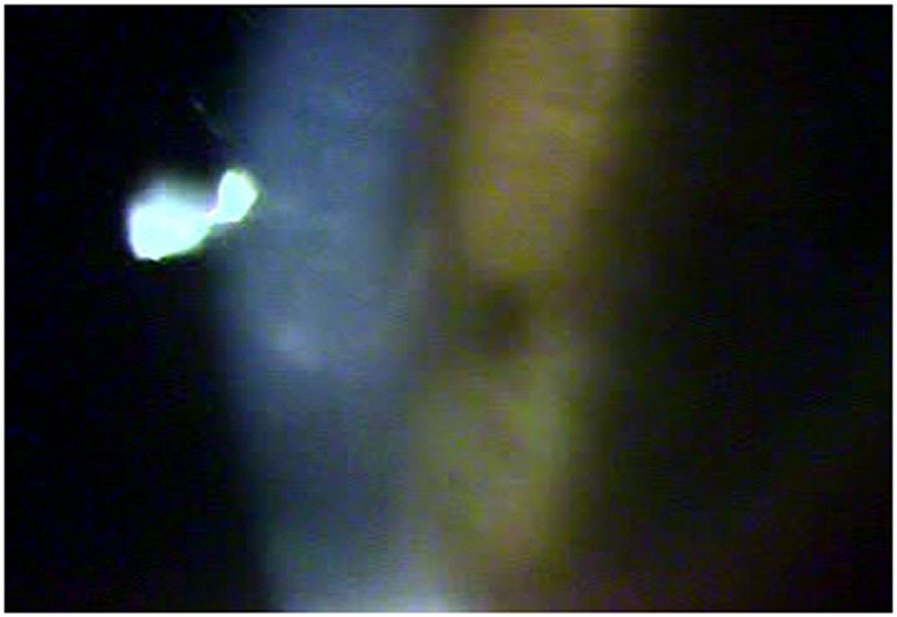
Descemet’s membrane rupture

**Fig. 3:**
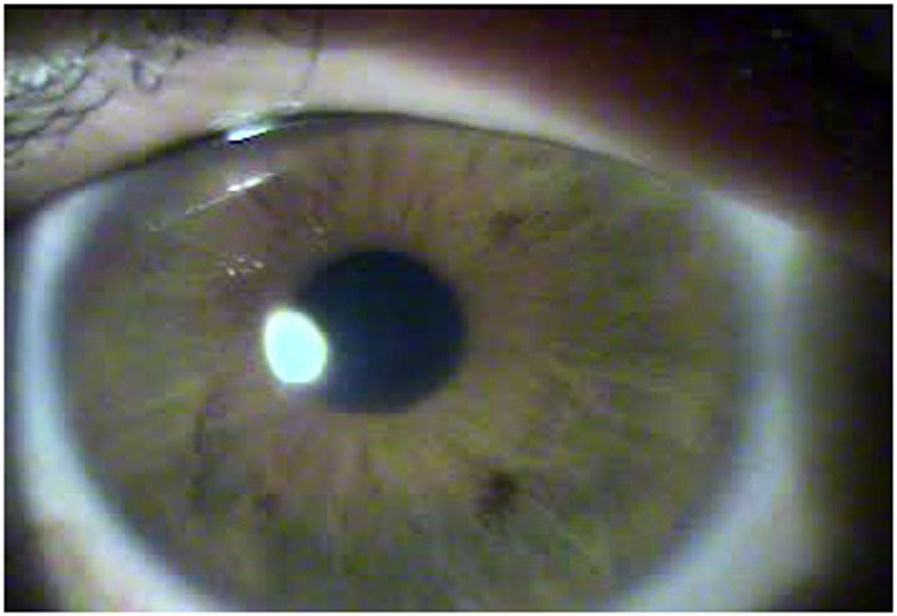
Complete resolution of the condition

LE biomicroscopy showed a clinical condition of acute corneal hydrops, with Descemet’s membrane rupture in the central area, approximately 2 mm horizontally and 4 mm vertically, and associated stromal edema, with no signs of inflammation in the anterior chamber (Fig. 2). Her VA was 20/500 without correction in the LE and 20/20 in the RE with an RCL. The RE was normal, with a well-adjusted RCL. Treatment with antibiotic eye drops and associated corticosteroids was initiated, and the patient returned 1 week later with no complaints about the LE and showing complete resolution of the corneal hydrops (Fig. 3).

**Fig. 4:**
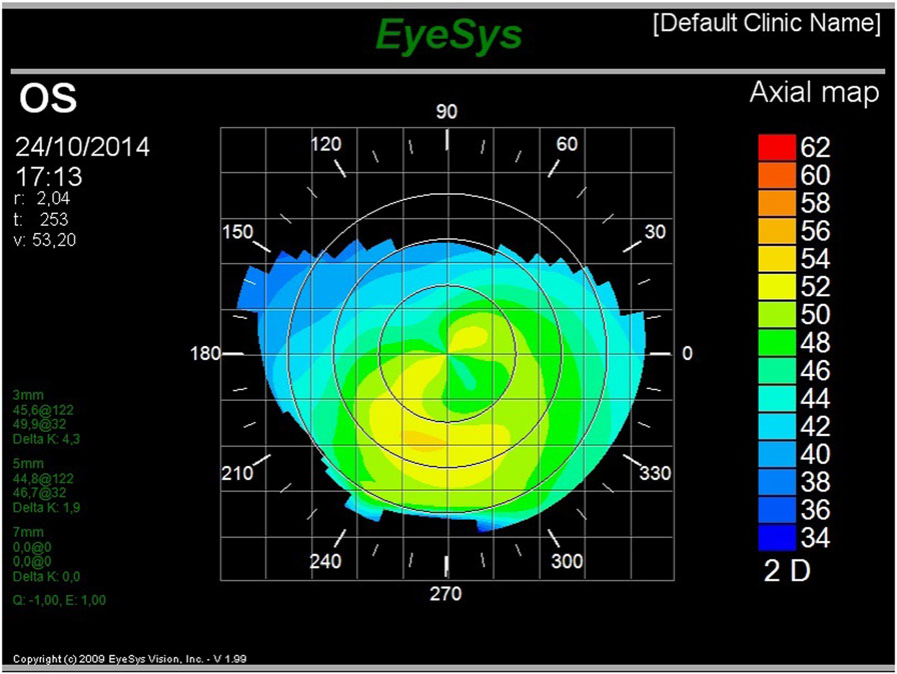
Left eye corneal topography showing resolution of the condition

LE corneal topography showed flattening of the maximum corneal curvature from 54.7 diopters to 53.20 diopters (Fig. 4). The patient showed a VA of 20/20 in both eyes with RCLs.

## Discussion

Hormonal effects, which remain poorly understood, are among the factors involved in keratoconus. Studies [3, 4] have shown that estradiol stimulates the production of matrix metalloproteinases and the production or activation of collagenolytic enzymes, which may be responsible for collagen weakening. Furthermore, estrogen increases the release of prostaglandins and activates collagenase. These events cause collagen degradation and therefore decreased corneal rigidity [3]. Thus, hormonal factors are related to the onset of keratoconus, and increased estrogen (which occurs during pregnancy) is related to its progression [4]. This case was followed for 9 months after performing CXL, and the lack of keratoconus progression during pregnancy was confirmed by corneal topography.

One of the most common complications of keratoconus is acute corneal hydrops, which is typically corneal edema resulting from the rupture of Descemet’s membrane and endothelium [5]. Continuous intrastromal buildup of aqueous humor causes the separation of collagen lamellae and formation of fluid-filled clefts/cysts [6]. The adjacent endothelium becomes hypertrophied in the area of rupture of Descemet’s membrane and determines the duration of resolution of the condition (within 5 to 36 weeks) [2].

In this study, the complete resolution of the patient’s acute corneal hydrops occurred in only 8 days. This resolution may have resulted from prior CXL, which promoted a photochemical reaction generating free radicals and oxygen radicals [7]. The free radicals catalyze the reaction and form covalent bonds between collagen molecules and microfibers [8] that stabilize the stroma and improve collagen structure, thereby hindering the formation and persistence of corneal stromal edema [7]. Furthermore, the biomechanical effect is related to an increase in corneal rigidity of approximately 328.9% [9]. Surprisingly, the patient progressed without haze and with 20/20 VA with RCLs, which was confirmed by a slit-lamp evaluation and the Snellen test.

## Conclusions

The present case led us to the hypothesis that CXL increases anterior stromal resistance through covalent bonds between collagen fibers. This increase in anterior stromal resistance would obstruct the passage of aqueous humor after rupture of Descemet’s membrane. Furthermore, increased corneal hardening would favor keratoconus nonprogression during pregnancy. However, other studies are necessary to determine whether CXL accelerates the resolution of acute corneal hydrops.

## Abbreviations

CXL: Collagen cross-linking
LE: Left eye
RCL: Rigid contact lens
RE: Right eye
VA: Visual acuity
